# DAVS-NET: Dense Aggregation Vessel Segmentation Network for retinal vasculature detection in fundus images

**DOI:** 10.1371/journal.pone.0261698

**Published:** 2021-12-31

**Authors:** Mohsin Raza, Khuram Naveed, Awais Akram, Nema Salem, Amir Afaq, Hussain Ahmad Madni, Mohammad A. U. Khan, Mui-zzud- din

**Affiliations:** 1 Department of Computer Science, Bahria University, Islamabad, Pakistan; 2 Department of Electrical and Computer Engineering, COMSATS University Islamabad (CUI), Islamabad, Pakistan; 3 Department of Primary and Secondary Healthcare, Lahore, Pakistan; 4 Effat College of Engineering, Electrical and Computer Engineering Department, Effat University, Jeddah, Saudi Arabia; 5 RF and Antenna Research Group, RMIT University, Melbourne, Australia; 6 Department of Electrical Engineering, Namal Institute Mianwali, Mianwali, Pakistan; 7 Department of Computer Science, Khwaja Fareed University of Engineering and Information Technology, Rahim Yar Khan, Pakistan; Vietnam National University, VIET NAM

## Abstract

In this era, deep learning-based medical image analysis has become a reliable source in assisting medical practitioners for various retinal disease diagnosis like hypertension, diabetic retinopathy (DR), arteriosclerosis glaucoma, and macular edema etc. Among these retinal diseases, DR can lead to vision detachment in diabetic patients which cause swelling of these retinal blood vessels or even can create new vessels. This creation or the new vessels and swelling can be analyzed as biomarker for screening and analysis of DR. Deep learning-based semantic segmentation of these vessels can be an effective tool to detect changes in retinal vasculature for diagnostic purposes. This segmentation task becomes challenging because of the low-quality retinal images with different image acquisition conditions, and intensity variations. Existing retinal blood vessels segmentation methods require a large number of trainable parameters for training of their networks. This paper introduces a novel Dense Aggregation Vessel Segmentation Network (DAVS-Net), which can achieve high segmentation performance with only a few trainable parameters. For faster convergence, this network uses an encoder-decoder framework in which edge information is transferred from the first layers of the encoder to the last layer of the decoder. Performance of the proposed network is evaluated on publicly available retinal blood vessels datasets of DRIVE, CHASE_DB1, and STARE. Proposed method achieved state-of-the-art segmentation accuracy using a few number of trainable parameters.

## 1 Introduction

Early detection of potential blindness diseases is vital to treat their progression and avoid vision loss, for instance, Aging based Mocular Degeneration (AMD), Diabetic Retinopathy (DR) and Hypertension Retinopathy (HR) [1]. Similarly, timely detection of Hypoxemia and Glaucoma is useful for availing cost effective remedies. It is widely understood that these diseases impact the structure of retinal blood vessels [2]. Therefore, clinicians diagnose these diseases by observing the visible changes in the structure of blood vessels in retinal images [3, 4]. That is a cumbersome process and hence is not practically viable to perform on a larger scale owing to the limitation of skilled labour and timing consuming nature of the process.

Consequently, Computer-aided diagnostic (CAD) systems have taken a deep root in eye diagnosis owing to their fast processing and ability to scan through large datasets of fundus images [5–7]. These computerized techniques start by employing segmentation strategies to extract patterns of blood vessels [8, 9]. That is followed by the use of automated classifiers to evaluate and analyze the extracted vessels for detection of variations in the characteristics of blood vessels [10]. Thus, leading to automated diagnosis of the eye. In this regard, the role of computerized vessel segmentation strategies is vital because the classifier’s effectiveness in eye disease highly depends on the accuracy of the segmented vessels [11, 12].

Retinal vessel segmentation has attracted significant attention from engineers and scientists, resulting in a wide range of state of the art methods [13–19]. However, effective segmentation of retinal vessels is still an open problem due to various challenges which involve sharp variations in vessel size, shape, and orientation, not to mention the low intensity, branching, and vessel crossovers. Consequently, identification of vessels and differentiating those from irregularities (arising due to a disease or other similar phenomenon) is a difficult task. That is further aggravated by the presence of various types of noise and artifacts due to fundus imaging modalities.

Retinal vessel segmentation has attracted significant attention from engineers and scientists, resulting in a wide range of state of the art methods [13–19]. However, effective segmentation of retinal vessels is still an open problem due to various challenges which involve sharp variations in vessel size, shape, and orientation, not to mention the low intensity, branching, and vessel crossovers. Consequently, identification of vessels and differentiating those from irregularities (arising due to a disease or other similar phenomenon) is a difficult task. That is further aggravated by the presence of various types of noise and artifacts due to fundus imaging modalities.

Earlier, classical image segmentation strategies were tailored to detect and segment out vessel patterns. These techniques identify vessels based on width, size, shape and orientation of vessels and hence are referred to as unsupervised methods [14–16, 20–22]. However, these methods can only capture limited types of vessels due to sharp variations in their shapes and sizes. Moreover, these techniques can not fully comprehend and eradicate the problem of low illumination and poor contrast regions in retinal fundus images. Although, contrast enhancement techniques are used as a pre-processing step that partially address the issue but they intensify the noise or artifacts present in the image [11, 23] which led to the use of noise removal as an additional pre-processing step in some recent unsupervised methods [24, 25].

Supervised methods, on the other hand, use trained Support Vector Machines (SVM) [10, 26] and Neural Networks (NNs) [27, 28] to identify vessels based on learned features from fundus images. Compared to SVM, NN can model the interrelationship between features in a much better way that has led to their increased use in this regard.

Deep learning techniques, which employ multi-layered NNs, have particularly yielded much higher rates of accuracy albeit at high computational cost [29–31]. Traditional DNNs do well to learn the inherent structures within the image that allow them to recover the structure of vessels in a much better way when compared with classical techniques. Deep Neural Networks (DNNs) have the ability to learn inherent and deep structures within the retinal images from a large sized fundus image dataset, allowing the detection of fine vessels [32, 33]. For this purpose, deep learning based techniques employ CNNs to extract desirable features which are finally used to identify vessels. Moreover, deep features allow these techniques to move past the problem of noise and artifacts. However, the problem with these methods is their lack of robustness when detecting less significant or minor vessels. This problem is due to the loss of important information due to pooling operations that restrict their efficacy. Consequently, recent vessel segmentation techniques employ semantic segmentation, where each pixel is classified as a vessel or the background. That provides the high precision needed to detect tiny vessels, such as vessels consisting of only few pixels.

This work proposes a novel network architecture, namely Dense Aggregation Vessel Segmentation network (DAVS-Net), for robust semantic segmentation of retinal vessels that is capable of detecting minor vessels owing to its pixel wise segmentation operation. The proposed architecture employs dense concatenation block that permits immediate transfer of spatial information within layers leading to the identification of pixels from the desired class. In addition, we propose an encoder-decoder framework that allows faster convergence by directly transferring the edge information from initial layer of the encoder to the last layer of the decoder. Moreover, the proposed network requires only a few trainable parameters as apposed to a large number of trainable parameters required in existing methods because of low-quality retinal images with different image acquisition conditions and intensity variations. The proposed DAVS-Net achives state of the art performance that is demonstrated publicly available retinal blood vessels datasets of DRIVE, CHASE_DB1, and STARE.

This paper is organized to provide background of the problem in Section II post the introduction in Section I. The proposed methodology is discussed in Section III followed by the results and discussion in Section IV. Finally, conclusions and scope for future work are discussed in Section V.

## 2 Background and related work

Semantic segmentation is regarded as a fundamental application in computer vision where pixel-wise classification is performed for all the pixels present in the image. This approach is able differentiate between pixels belonging to objects and those belonging to the background leading to the detection of tiniest objects. Consequently, semantic segmentation is well suited for retinal vessel segmentation since detection of tiniest of vessels is vital for analysis and diagnosis of retinal disease.

The conventional deep learning-based methods [34] effectively learn structures of significant objects but lack robustness to identify the minor ones. Thus, the DNNs used for segmentation are not local enough in their operation and as a consequence, they do not classify each pixel for detection of a vessel leading to loss of minor and tiny vessels. Deep networks for vessel detection use many convolutional and pooling layers which cause vanishing gradient problems. This loss of spatial information degrades the overall performance of pixel-wise classification. To overcome the vanishing gradient problem residual networks (Res-Nets) [35] were introduced that used the residual skip connection to improve the performance and manage the gradient during the training process. However, Res-Nets caused the feature transfer impedance problem that was later covered by Dense-Net [36] through deep feature concatenation.

Another factor affecting the segmentation of tiny vessels is the compromised quality of fundus images typically caused by the limitation of varying acquisition conditions. Hence, robust segmentation of retinal vessels is an open problem with a focus on the detection of minor vessels which provide critical additional information for automated eye diagnosis.

## 3 Proposed methodology

In this work, we propose DAVS-Net architecture for robust semantic segmentation of retinal vessels from the fundus image by effectively capturing the minor vessels along with the significant ones. Proposed architecture seeks to address limitations of traditional deep learning techniques which employ a number convolutional layers followed by pooling operations that means local information about each and every pixel is not readily available. As a result, these networks work well to detect significant vessels although it means that identification of minor vessels becomes challenging. This issue needs special attention as detection of smaller vessels is critical to accurate eye disease diagnoses.

To address this issue, proposed dense aggregation network, for semantic segmentation of retinal vasculature, feeds on the desirable properties of the DenseNet [36] that is famous for its classification performance. That is because of the use of the dense concatenation which alleviates the feature latency problems and provide higher accuracy compared to ResNet [35], and VGG [37]. Considering the effectiveness of feature concatenation benefits, the connectivity of the DAVS-Net is inspired from Dense-Net. The key differences of proposed DAVS-Net and DenseNet are mentioned in Table 1.

**Table 1:** Difference between DenseNet and proposed DAVS-Net.

DenseNet	DAVS-Net
DenseNet is a classification network with fully connected layers	DAVS-Net is a semantic segmentation network which does not use fully connected layer to operate in fully convolutional manner
DenseNet does not use any upsampling (decoder)	DAVS-Net is an encoder-decoder network
DenseNet used many dense blocks (E.g five dense block for ImageNet dataset)	DAVS-Net is just using 3 dense block is encoder and 3 dense blocks for decoder
In each dense block DenseNet use four convolutional layers	DAVS-Net net use just two convolutions in each dense block
DenseNet does not use unpooling layers, so it does not transfers the pooling indices	DAVS-Net uses unpooling layers in combination with pooling layers, so it transfers the pooling indices to decoder
DenseNet uses global average pooling in the end of the network	DAVS-Net use max-pooling layer after each dense block

### 3.1 Overview of proposed architecture

**Fig 1:**
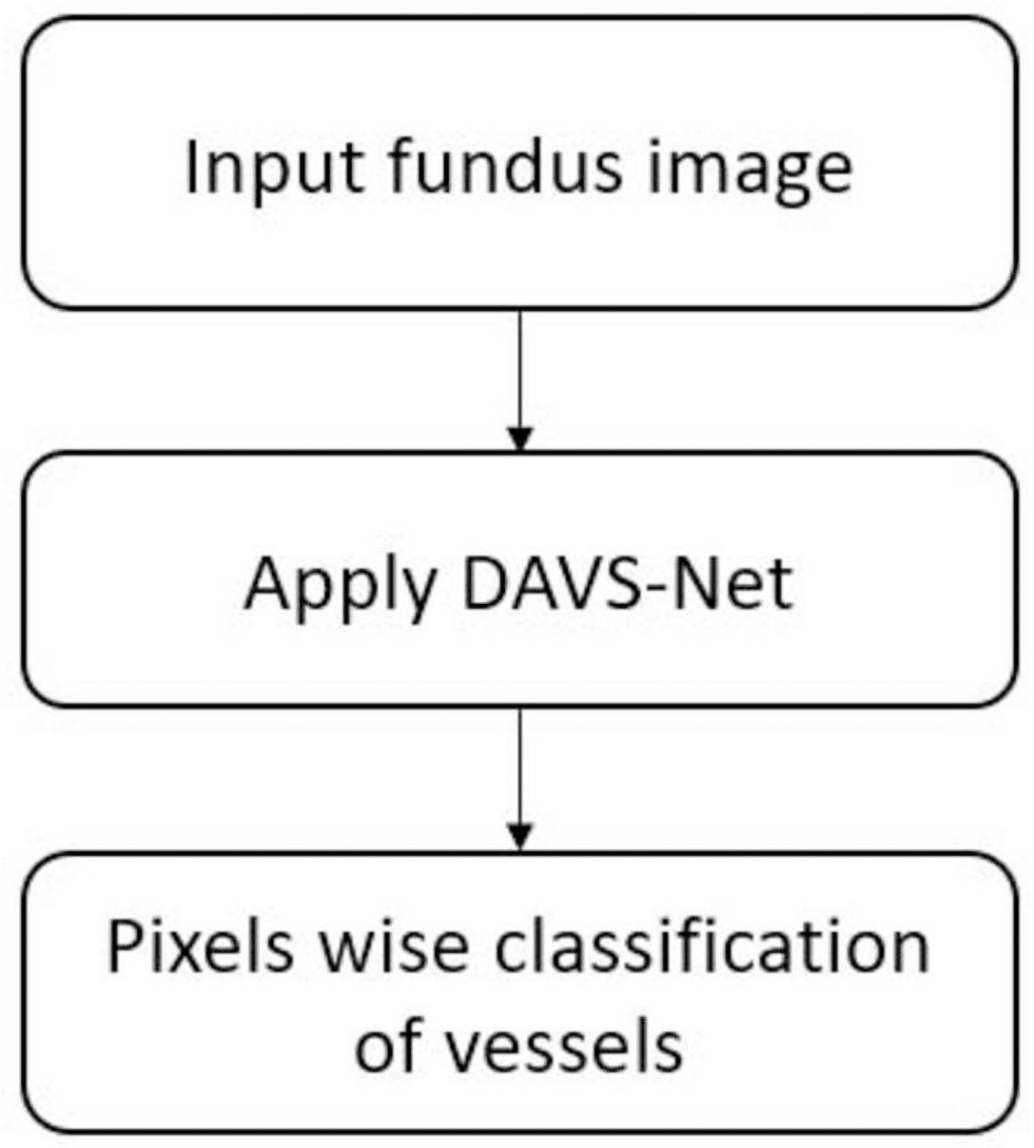
Flow diagram of the proposed method.

The proposed DAVS-Net is designed to take advantage of the deep feature that allows to skip the pre-processing and does not require any enhancement in the quality of the input image. That is because the deep feature allows to allows to import and combine high-frequency information from the corresponding layers thus circumventing the imaging artifacts and bring to light the main features of the image. Owing to that, DAVS-Net is capable of detecting vessel pixels from noisy and low-quality images and non-uniform illumination. The overall principle of the proposed method is summarized in Fig 1. Moreover, the pixel wise segmentation operation and the marking of blood vessels yields the much needed accuracy for vessel detection. The output of the proposed method is a binary image with a representation of ‘1’ for vessel pixels and ‘0’ for the background.

### 3.2 Working principle of the DAVS-Net

Proposed DAVS-Net considers dense connections as a means to boost accuracy of the semantic segmentation. To this end, the problems faced by traditional deep learning techniques are addressed using its following key features:

1. Fewer convolutional layers and pooling layers are used to reduce the spatial information loss.
2. Dense concatenation of the features is used within the dense block to enable the network for providing immediate spatial information transfer between the layers.
3. The edge information transfer from the first layers of the encoder to the last layer of the decoder is used for faster convergence of the network.

**Fig 2:**
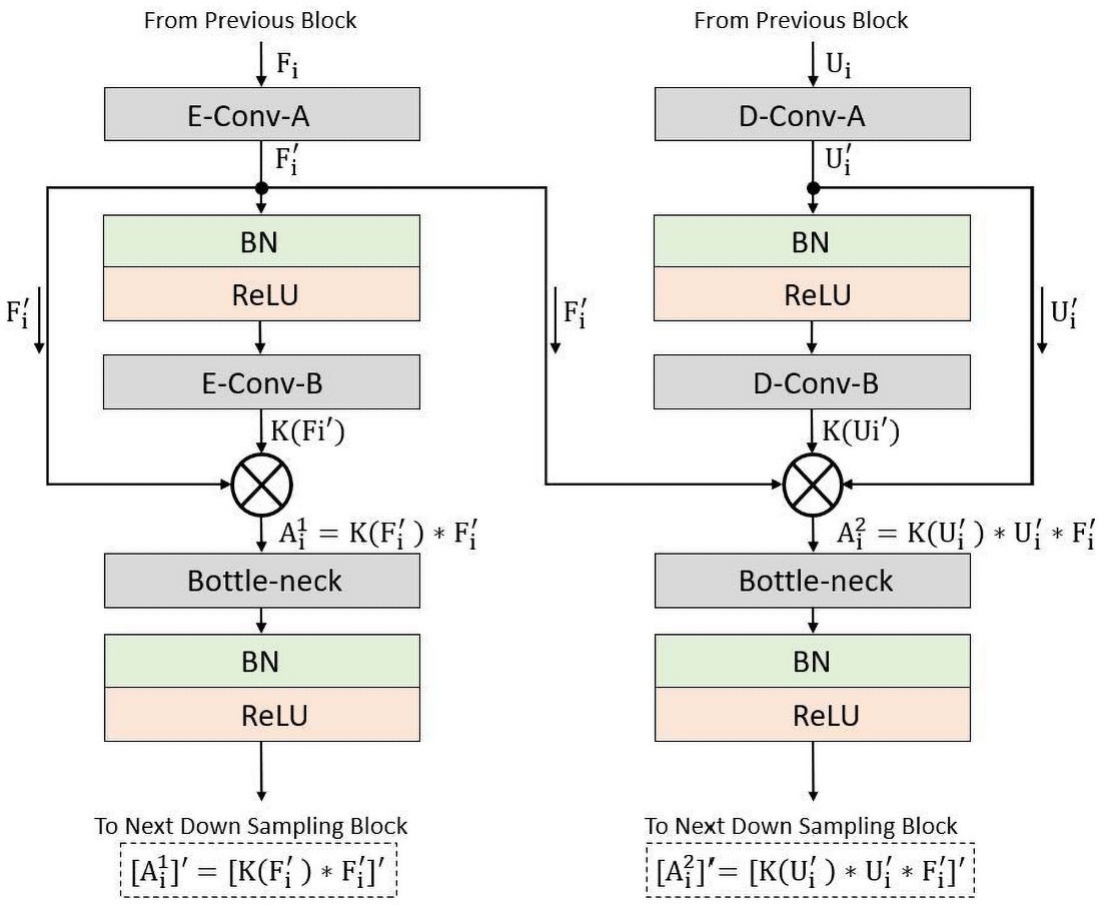
Connectivity principle of DAVS-Net.

The connectivity principle of DAVS-Net is demonstrated in Fig 2 that presents the layout of the deep feature concatenation for the candidate encoder-decoder block.

The encoder consists of three dense blocks with two convolutional layers in each block. Similar structure is used for the decoder as well. We describe both encoder and decoder in detail in Section 3.3 and 3.4. Here, we discuss the connectivity of principle of the proposed DAVS-Net (as given in Fig 2) that leads to formulation of deep feature.

Specifically, the dense block of the encoder, shown on the left side of Fig 2, receives an input feature *F*_*i*_ while the dense block of the decoder, depicted on the right side of Fig 2, receives an input feature *U*_*i*_. The feature $F_{i}^{{}^{\prime }}$ is obtained after two convolutional operations, namely *E-Conv-A* and *E-Conv-B*. The spatial loss is recovered by deep feature concatenation of these two convolutional layers. The dense feature $A_{i}^{1}$ is obtained by concatenating the feature of the outputs $F_{i}^{{}^{\prime }}$ and $K(F_{i}^{{}^{\prime }})$ of *E-Conv-A* and *E-Con-B*, as given below:

$$\begin{array}{c} A_{i}^{1}=K\left(F_{i}^{{}^{\prime }}\right)*F_{i}^{{}^{\prime }}, \end{array}$$ (1)

where ‘*’ denotes the depth-wise concatenation.

We next employ a bottleneck layer, termed *Bottle-Neck*, to limit number of channels after a Batch Normalization (BN) and a Rectified Linear Unit (ReLU) operations that results in the feature ${\left[A_{i}^{1}\right]}^{{}^{\prime }}$, as follows

$$\begin{array}{c} {\left[A_{i}^{1}\right]}^{{}^{\prime }}={\left[K\left(F_{i}^{{}^{\prime }}\right)*F_{i}\right]}^{{}^{\prime }}. \end{array}$$ (2)

Similarly, the decoder applies a convolution on the input *U*_*i*_ through the convolutional layer *D-Conv-A* resulting in feature $U_{i}^{{}^{\prime }}$. This feature *U*_*i*_ is then fed to the second convolutional layer *D-Conv-B* resulting in the feature $K(U_{i}^{{}^{\prime }})$. The spatial loss is recovered by concatenating the deep feature from these two convolution layers and the third feature $F_{i}^{{}^{\prime }}$ that comes from the encoder by an external dense path. Thus, the dense feature $A_{i}^{2}$ is an enriched feature by the concatenation of three features of the outputs *D-Conv-A*, *D-Conv-B*, and *E-Conv-A* as given below:

$$\begin{array}{c} A_{i}^{2}=K\left(U_{i}^{{}^{\prime }}\right)*U_{i}^{{}^{\prime }}*F_{i}^{{}^{\prime }} \end{array}$$ (3)

Just like in encoder, the increase in the number of channels for $A_{i}^{2}$ feature may lead to memory consumption that is resolved through the *Bottle-Neck* layer after BN and ReLU operations yielding to the feature $A_{i}^{2}$, as follows:

$$\begin{array}{c} {\left[A_{i}^{2}\right]}^{{}^{\prime }}={\left[K\left(U_{i}^{{}^{\prime }}\right)*U_{i}^{{}^{\prime }}*F_{i}^{{}^{\prime }}\right]}^{{}^{\prime }}. \end{array}$$ (4)

**Fig 3:**
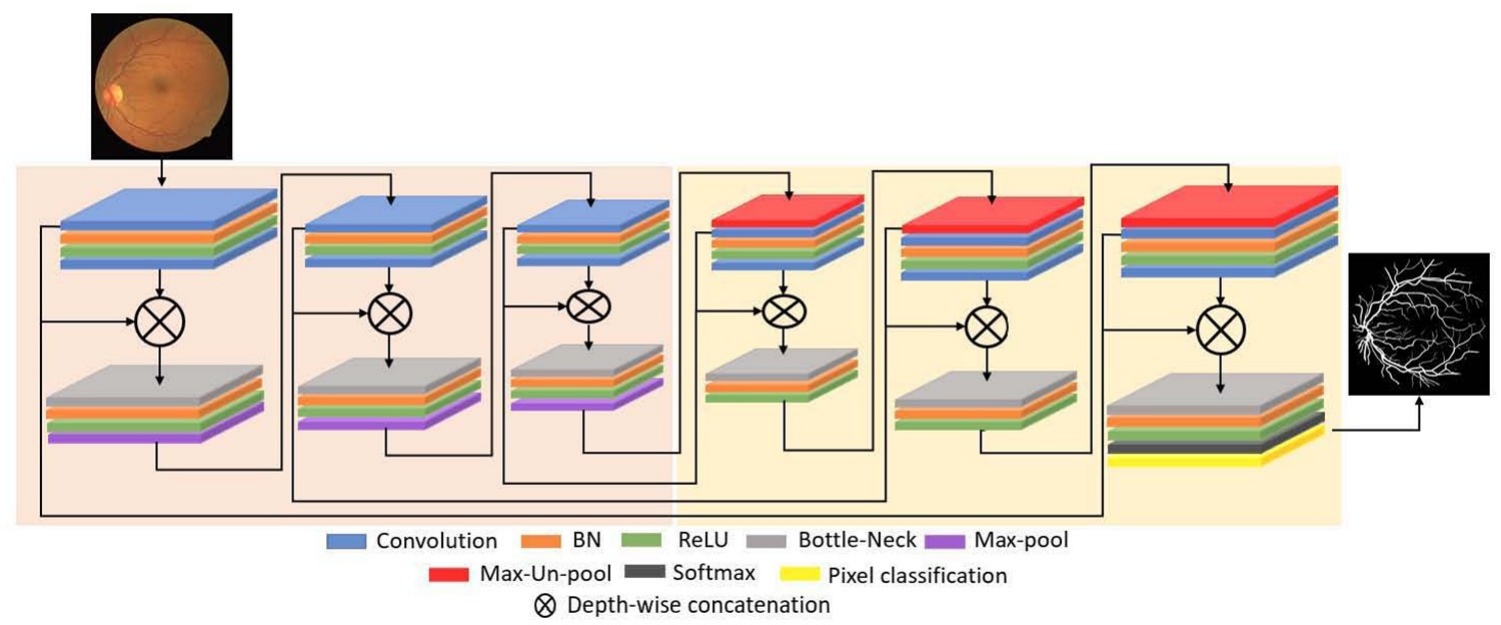
Architecture of DAVS-Net used for vessel segmentation in our work.

Now, comparing both ${\left[A_{i}^{1}\right]}^{{}^{\prime }}$ and ${\left[A_{i}^{2}\right]}^{{}^{\prime }}$, although both are empowered features by dense connectivity but the ${\left[A_{i}^{2}\right]}^{{}^{\prime }}$ is the resultant feature of three features concatenation which also includes the important edge information. Owing to that enrichment, the proposed DAVS-Net is able to perform finer segmentation without any prior need for preprocessing. To ensure the segmentation of small objects, feature enhancement is done at dense block level as shown in Fig 3, that presents the complete architecture with the dense feature concatenation. The DAVS-Net is keeping the feature map size before upsampling at 80 × 80 for an input image of 640×640, that is enough to represent the valuable features for vessel segmentation.

### 3.3 DAVS-Net encoder

DAVS-Net is a densely connected fully convolutional network that uses a total of 6 dense blocks for both encoder and decoder as shown in Fig 3. The encoder consists of three dense blocks with each block containing two convolutional layers. Each encoder dense block starts with a convolutional layer and ends with a pooling layer that is used to reduce the size of the feature map. As an example, the first encoder dense blocks is with two convolutions of 64 channels, and the output of both convolutions are merged by a depth-wise concatenation layer generating 128 channels.

The concatenation layer leads to increase in depth of the feature map that requires more memory as well as processing power. The issue is addressed through bottleneck layer that reduces memory consumption by selecting higher minibatch size in each dense block which results in limiting the channels after the concatenation. Moreover, a constant convolution operation is required to segment the image using a convolutional neural network (CNN). Consequently, the DAVS-Net encoder performs the constant convolutional operation on the image and the feature. That travels through the network in a feed-forward fashion until the image is represented by the tiny features.

Another problem with CNN is that max-pooling operation (post convolution) causes spatial information loss. In DAVS-Net, loss of the useful information is covered by the deep feature concatenation. Thus, in the proposed architecture, the encoder is composed of three dense block with 6 convolution layers and three max-Pool layers and the final feature map is 80×80 for a 640×640 input image. The DAVS-Net encoder structure in terms of the dense block is listed in Table 2, which describes the feature empowerment inside each encoder dense block and shows how the bottleneck layer reduces the depth of the feature map. The number of trainable parameters is also shown in the table for the layers in the encoder.

**Table 2:** DAVS-Net encoder-decoder I/O feature map sizes. Where EDB, EDB-C, EDB-Cat, DDB, DDB-C, DDB-Cat represent encoder dense block, encoder dense block convolution, encoder dense block concatenation, decoder dense block, decoder dense block convolution, decoder dense block concatenation, respectively. The layer with $\hat{/}$ shows that layer includes rectified linear unit (ReLU), and batch normalization (BN) after.

Dense Block	Layer/Size	Filters	Layer O/P	Parameters
EDB1	EDB1-C1$\hat{/}$3 × 3 × 3 to (DDB1-C1) & EDB1-Cat	64	640 × 640 × 64	1792 + 128
	EDB1-C2 /3 × 3 × 64 to EDB1-cat	64	640 × 640 × 64	36,928
	EDB1-Cat (EDB1-C1 * EDB1-C2)	-	640 × 640 × 128	-
	E-Bneck-1$\hat{/}$1 × 1 × 64	64	640 × 640 × 64	8256 + 128
	Pool-1	-	320 × 320 × 64	-
EDB2	EDB2-C1$\hat{/}$3 × 3 × 64 to (DDB2-C1) & EDB2-Cat	128	320 × 320 × 128	73,856 + 256
	EDB2-C2 /3 × 3 × 64 to EDB2-Cat	128	320 × 320 × 128	147,584
	EDB2-Cat (EDB2-C1 * EDB2-C2)	-	320 × 320 × 256	-
	E-Bneck-2$\hat{/}$1 × 1 × 128 × 64	64	640 × 640 × 64	8256 + 128
	Pool-2	-	160 × 160 × 128	-
EDB3	EDB3-C1$\hat{/}$3 × 3 × 64 to (DDB3-C1) & EDB3-Cat	256	160 × 160 × 256	295,168 + 512
	EDB3-C2 /3 × 3 × 64 to EDB3-Cat	256	160 × 160 × 256	590,080
	EDB3-Cat (EDB3-C1 * EDB3-C2)	-	160 × 160 × 512	-
	E-Bneck-3$\hat{/}$1 × 1 × 256	64	160 × 160 × 256	131328 + 512
	Pool-3	-	80 × 80 × 256	-
DDB3	Unpool-3	-	160 × 160 × 256	-
	DDB3-C1$\hat{/}$3 × 3 × 256 to DDB3-Cat	256	160 × 160 × 128	590,080 + 512
	DDB3-C2 /3 × 3 × 256 to DDB3-Cat	64	160 × 160 × 640	295,040
	DDB3-Cat (DDB3-C1 * DDB3-C2 * EDB3-C1)	-	160 × 160 × 640	-
	D-Bneck-1$\hat{/}$1 × 1 × 640	128	160 × 160 × 128	82048 + 256
DDB2	Unpool-2	-	320 × 320 × 128	-
	EDB2-C1$\hat{/}$3 × 3 × 64 to (DDB2-C1) & EDB2-Cat	128	320 × 320 × 64	147,584 + 256
	EDB2-C2 /3 × 3 × 64 to EDB2-Cat	64	320 × 320 × 128	73,792
	DDB2-Cat (DDB2-C1 * DDB2-C2 * EDB2-C1)	-	320 × 320 × 320	-
	D-Bneck-2$\hat{/}$1 × 1 × 320	64	320 × 320 × 64	20544 + 128
DDB1	Unpool-1	-	640 × 640 × 64	-
	DDB1-C1$\hat{/}$3 × 3 × 64 to DDB1-Cat	64	640 × 640 × 64	36,928 + 128
	DDB1-C2 /3 × 3 × 64 to DDB1-Cat	2	640 × 640 × 2	1,154
	DDB1-Cat (DDB1-C1 * DDB1-C2 * EDB1-C1)	-	640 × 640 × 130	-
	D-Bneck-3$\hat{/}$1 × 1 × 130	2	640 × 640 × 2	262 + 4

### 3.4 DAVS-Net decoder

The decoder in DAVS-Net employs the reverse operation to the encoder as shown in Fig 3, whereby each dense block starts with an Max-Unpool layer which is responsible for gradually increasing the size of the feature map. After each unpooling layer, two convolutions follow the same concatenation and bottleneck principle. The depth-wise concatenation layer in each decoder block receives three inputs from first convolution, second convolution and direct information from the outer dense connection of the respective encoder block. The outer dense paths start from the first convolutional layer of the encoder dense block and terminate at the concatenation layer of each decoder dense block. These outer dense paths provide the immediate edge information from encoder to decoder to reduce the latency.

Specifically, the DAVS-Net decoder receives an input of 80×80-pixel from the encoder and provides the final feature map of the size equal to the size of input image. The bottleneck layer in each decoder block is used to reduce the number of channels to avoid memory issues. The last bottleneck layer in the decoder (third decoder dense block) is responsible for reducing the depth of the feature map. That also works as a class mask layer whose number of channels is equal to the number of classes.

This study is based on two classes “Vess” and “BG” representing vessel pixel and backgroud pixels; therefore, the number of channels in the last bottleneck layer is set at 2. The DAVS-Net pixel classification layer in combination with ‘Softmax’ function is responsible to assign a label to each pixel in the image from the available class based on prediction. Table 2 provide the layer layout of the DAVS-Net decoder with respective feature map sizes.

Table 3 presents the architectural differences of the proposed method with similar state-of-the-art networks. That essentially demonstrates that proposed architecture requires less convolution operations reduced channel depth as compared to some of other state of the architectures. Additionally, we also utilize dense connectivity, unpooling and bottleneck layers to further enhance the architecture of the proposed DAVS-Net over the comparative state of the art techniques.

**Table 3:** Comparison of architectural differences with similar state-of-the-art networks.

Model	Other architecture	DAVS-Net
**SegNet** [35]	Overall 26 convolutional layers	Overall 12 convolutional layers
	No residual or dense connectivity	Dense connectivity is used
	First two blocks have two convolutional layers while other include three convolutional layers	Only two convolutions in each block
	The block with channel depth-512 is used twice	Did not use Channel depth-512
**U-Net** [38]	Overall 23 convolutional layers	Overall 12 convolutional layers
	Up convolutions in decoder	Unpooling layers in decoder
	No dense connectivity within encoder/decoder (just dense connectivity encoder to decoder)	Inner and outer dense connectivity for both encoder and decoder
	1024 chandel-depth is used in bridge which involve many trainable parameters	maximum channel depth-256
	Use cropping	Dis not use cropping
**Vess-Net** [39]	Overall 16 convolutional layers	Overall 12 convolutional layers
	Based on residual connectivity	Based on dense connectivity
	First convolutional block missing with feature empowerment connectivity	Each convolutional layer is connected with dense empowerment
	No bottleneck layers are employed	Bottleneck layers are used to control number of channels
	10 residual paths	12 dense paths
**Dense-U-Net** [40]	Overall 89 convolutional layers	Overall 12 convolutional layers
	Overall 10 dense blocks are used in both encoder and decoder	Overall 6 dense blocks are used in both encoder and decoder
	unpooling layers are not utilized	Pooling and unpooling layers are used in combination
	Eight convolutions in each dense block	Two convolutions in each dense block
	Maximum channel depth-512	Maximum channel depth-256
	4 bottleneck layers are used in just encoder	6 bottlenck layers are used in both encode and decoder

## 4 Detection of diabetic and hypertensive retinopathy

It is mentioned in [41] that both diabetic and hypertensive retinopathy cause changes in retinal vessels. The diabetic retinopathy can swell the retinal vessels or even can create new blood vessels (increase in the vessel pixels), where the hypertensive retinopathy causes the shrinkage of retinal blood vessels (decrease in number of vessel pixels). The accurate segmentation of these vessels can provide an opportunity to detect changes in the retinal vessels (increase or decrease in number of vessels). This increase or decrease in number of vessel pixels can be used for diagnostic purposes for analysis of diabetic and hypertensive retinopathy. The disease progression can also be analyzed by comparing the masks of successive visits.

## 5 Experimental results

The experiments were conducted on a machine with Intel(R) Xeon(R) W-2133 CPU 3.60GHz processor, 96GB RAM, and Nvidia 2080TI GPU. For our implementation, the MATLAB was employed. We employed the ADAM optimizer with an initial learning rate of 1*e*^−3^, an exponential decay rate of 0.9, and mini-batch size of 10 images. The proposed DAVS-Net is trained from the scratch without weight initialization or migration from other frameworks. A weighted cross-entropy loss is used as an objective function for training in all of our experiments. This decision is based on the fact that the “background” pixels in each retinal image heavily outnumber the “foreground” pixels. We use median frequency balancing to calculate class association weights here [34].

Because the retinal vessel segmentation data sets used here are quite small in size, we used data augmentation to generate enough data for training. We used rotation and contrast enhancement to enhance the data. Each training image is rotated by 1 degree for the rotations. The contrast has been improved by randomly increasing and decreasing the image brightness. This results in 7600 images for the DRIVE and CHASE DB data sets, as well as 7000 images for each of the STARE data’s leave-one-out trails.

### 5.1 Materials

We have evaluated the performance of our proposed method on the basis of the following three fundus retinal image datasets which are publically available.

1. STARE: A group of twenty fundus images collected in the USA [41].
2. DRIVE: A collection of retinal images obtained from aged diabetic patients in Netherland [42].
3. CHASE_DB1: A collection of retinal fundus images based on fourteen pediatric subjects [43].

Segmentation of blood vessels is performed on retinal images in DRIVE dataset using manual procedure. In comparison of the three datasets, there is a binary mask revealing FOV for DRIVE dataset but it is not available for STARE and CHASE_DB1. For the STARE and CHASE_DB1 datasets, binary masks are manually generated by well-known techniques [44]. DRIVE and CHASE_DB1 have their individual and distinct training and testing datasets. In STARE, two subsets of randomly selected images are taken for training and testing purpose. As given in literature, a “leave-one-out” method is commonly implemented to separate training and testing sets [44]. In this method, a model is trained on ‘n-1’ samples and tested on the remaining sample to avoid overlapping. This process is iterated for ‘n’ times to complete the dataset, “leaving out” each sample at least once for the whole dataset. We have implemented this “leave-one-out” method for STARE dataset to train the model. Details of three selected datasets in our experiments are summarized in the Table 4.

**Table 4:** Summary of datasets used in the experiments.

Dataset Name	Training Set	Test Set	Dataset Size	Dimension (pixels)
STARE	10	10	20	700 605
CHASE_DB1	20	8	28	999 960
DRIVE	20	20	40	565 584

### 5.2 Evaluation criteria

Models for vessel segmentation are actually binary classifiers that necessarily differentiate vessels from the background for the given set of retinal fundus images. Performance of these segmentation classifiers is evaluated with “ground truth” images marked by ophthalmologists. Based on the following four parameters, we utilized the three metrics given in equations 5, 6, and 7 [36], for the performance evaluation of our proposed system.

1. True Negative (TN): Classifier correctly found as non-vessels,
2. False Positive (FP): Classifier incorrectly found vessels which are actually non-vessels,
3. True Positive (TP): Classifier correctly found as vessels,
4. False Negative (FN): Classifier incorrectly found non-vessels which are actually vessels.

$$\begin{array}{c} Sp=\frac{TN}{TN+FP} \end{array}$$ (5)

$$\begin{array}{c} Se=\frac{TP}{TP+FN} \end{array}$$ (6)

$$\begin{array}{c} Acc=\frac{TP+TN}{TP+FN+TN+FP} \end{array}$$ (7)

where *Sp*, *Se*, and *Acc* are representing the specificity, sensitivity, and accuracy, respectively. Accuracy is the ratio between correctly detected pixels (vessels and non-vessels) and the total pixels in the mask (FOV only). While specificity and Sensitivity demonstrate that how much accurately a model identifies the non-vessel and vessel pixels respectively. Furthermore, performance of the classifier is also assessed by some other parameters such as area under the Receiver Operating Characteristic (ROC), Area Under the Precision-Recall Curves (AUCPR), and False Positive Rate (FPR). Whenever, we have imbalanced distribution, ROC is a feasible assessing parameter for the classification of objects [45]. The AUC and AUCPR measures are used to analyze the objective efficiency of classification.

### 5.3 Comparison with state-of-the-art

**Fig 4:**
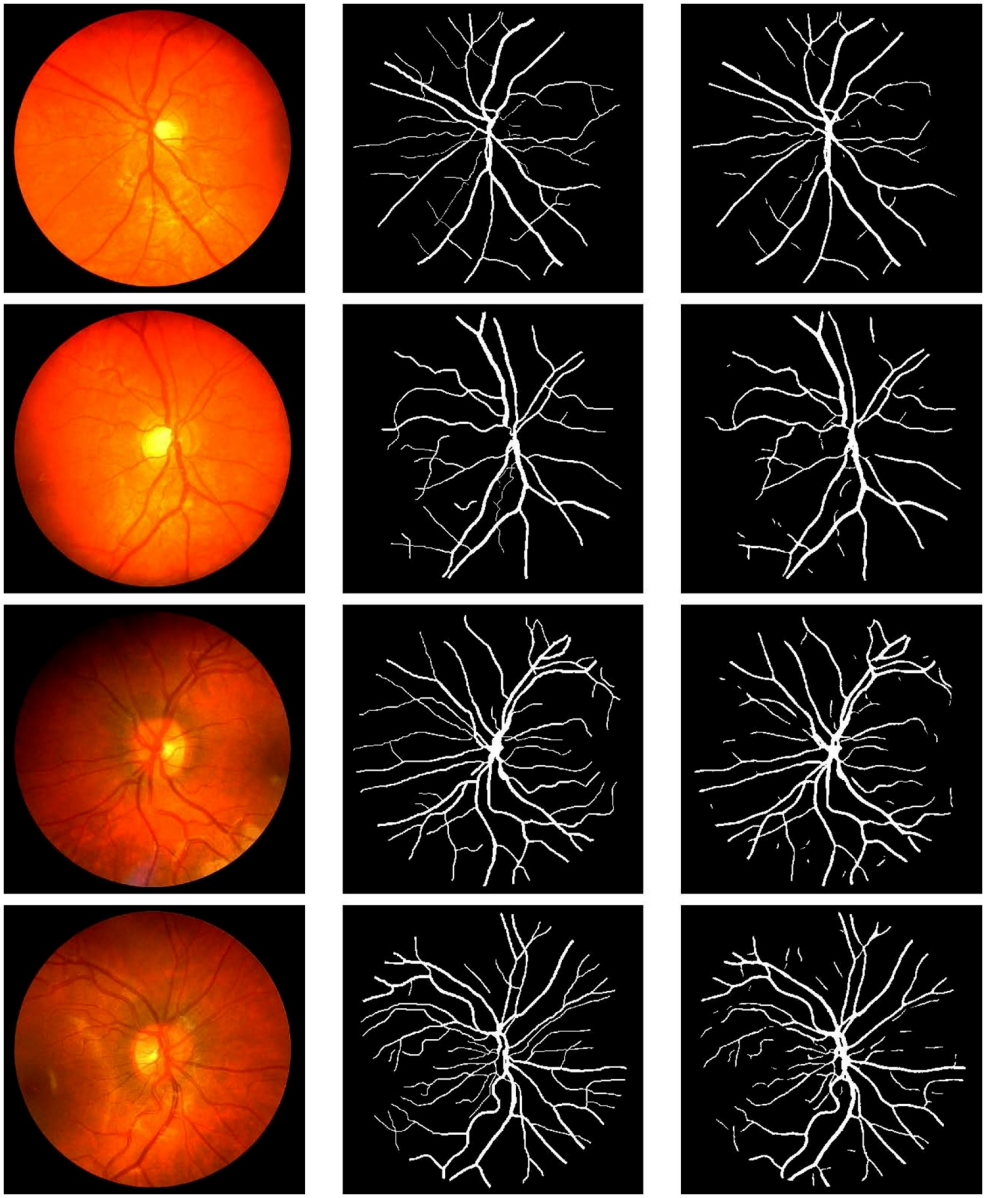
Visual results on the CHASE_DB1 dataset. From left-to-right: input images, ground truth, result obtained by our proposed method.

**Fig 5:**
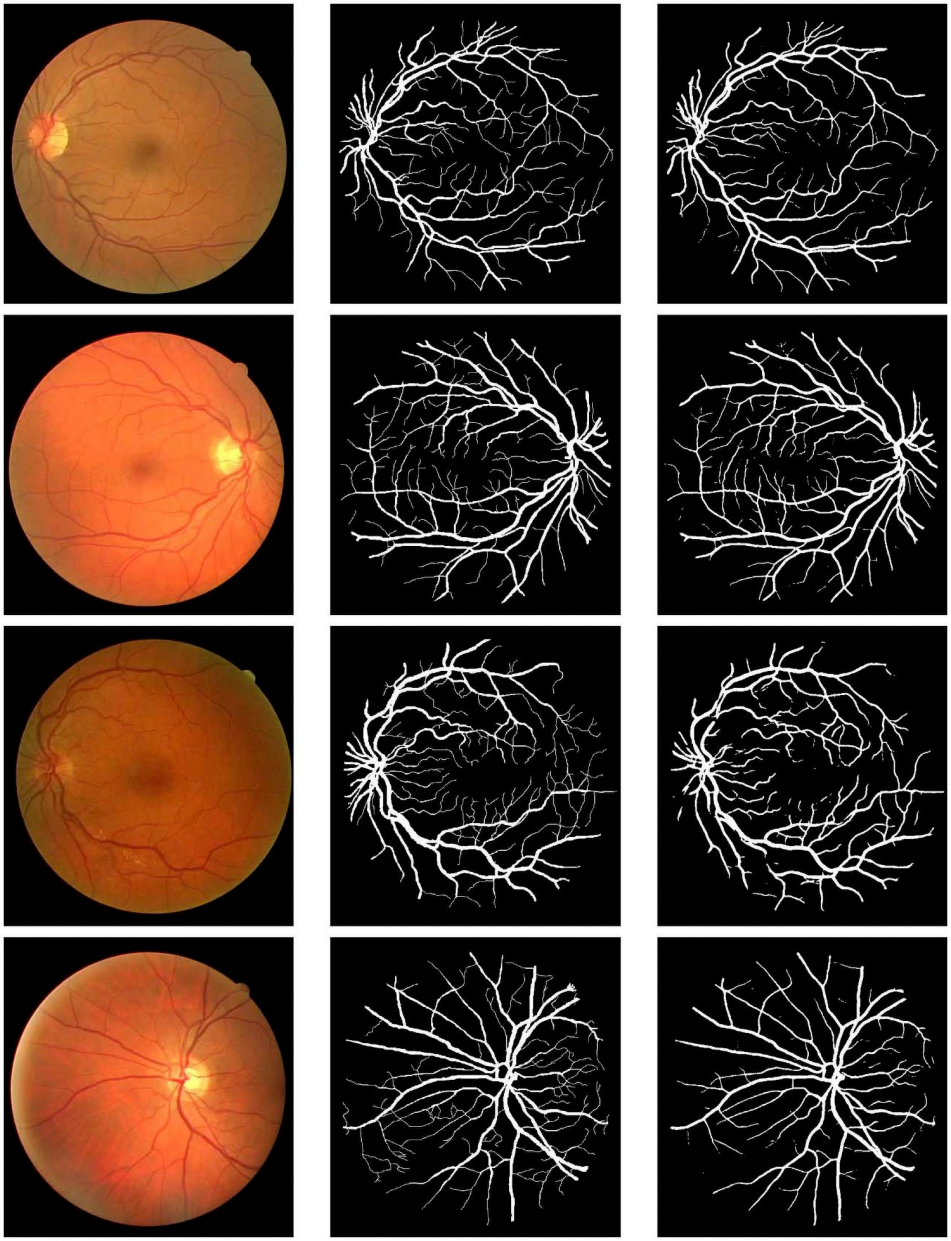
Visual results on the DRIVE dataset. From left-to-right: input images, ground truth, result obtained by our proposed method.

**Fig 6:**
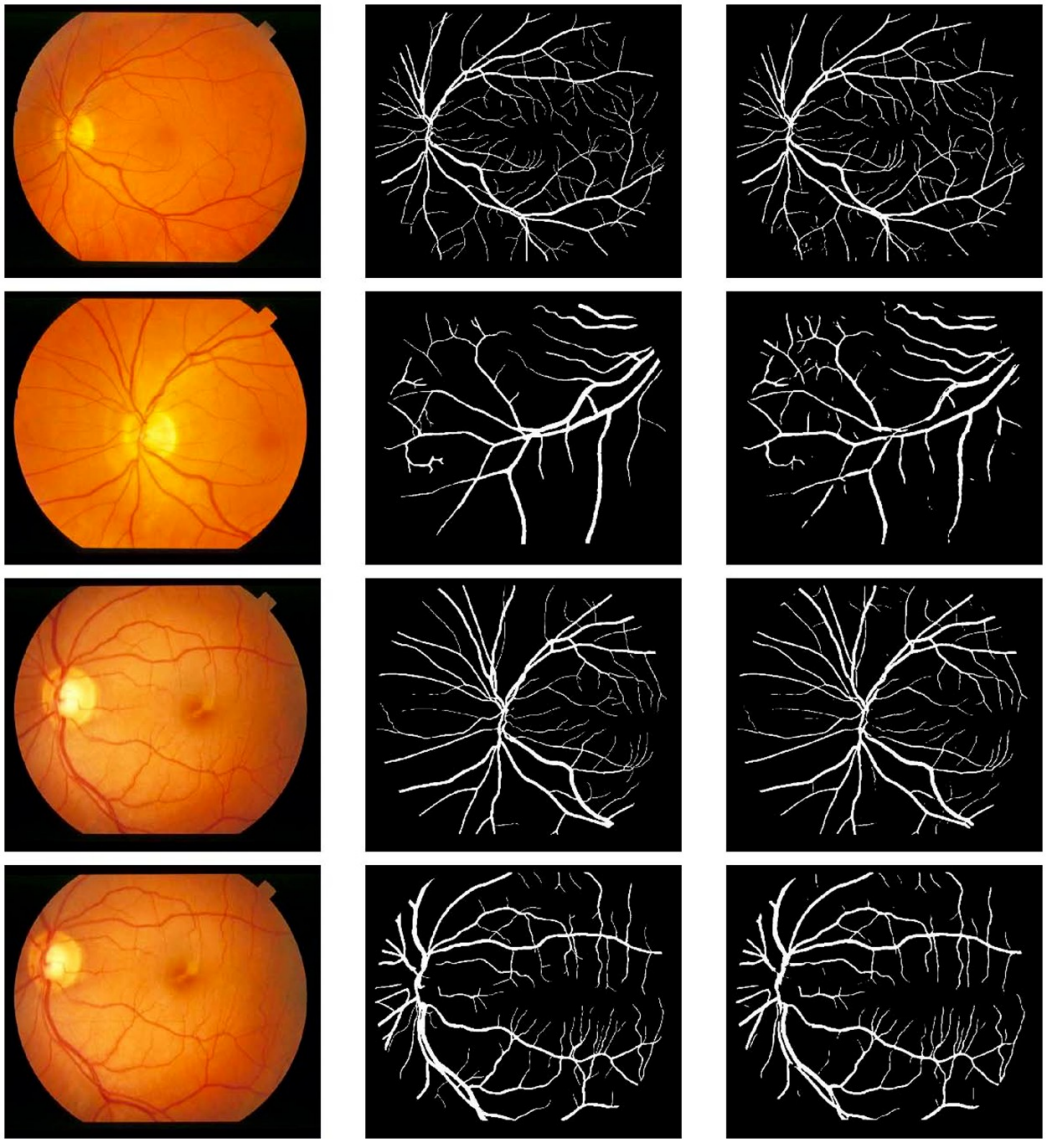
Visual results on the STARE dataset. From left-to-right: input images, ground truth, result obtained by our proposed method.

The visual results of our simulation on the three datasets are shown in Figs 4–6, respectively. In each figure, moving from left to right, the first column shows the original images, the second column shows the ground truth images and the third column shows the segmented images.

To evaluate and compare our results with those of state-of-the-art models, we have presented and summarized the results in tabular forms. Results obtained by our simulation on CHASE_DB1 are compared in Table 5. As given in the table, dice and Jaccard Se, Sp and Accuracy of our models are 0.8144, 0.9843 and 0.9726 respectively.

**Table 5:** Performance comparison of our proposed model on CHASE_DB1 dataset with other existing models.

Method	Year	Se	Sp	Acc	AUC
Khawaja *et al* [8]	2019	0.7974	0.9697	0.9528	NA
Zhang *et al* [46]	2016	0.7626	0.9661	0.9452	0.9606
Arsalan *et al* [39] VessNet	2019	0.8206	0.9800	0.9726	0.9800
Jin *et al* [47]	2019	0.7595	0.9878	0.9641	0.9832
Yin *et al* [48]	2020	0.7993	0.9868	0.9783	0.9869
Wang *et al* [49]	2020	0.8186	0.9844	0.9673	0.9881
Segnet-basic [35]	2020	0.8190	0.9735	0.9638	0.9780
**Our Method**	2021	**0.8144**	**0.9843**	**0.9726**	**0.9855**

In Table 6, the results of our proposed model, implemented on DRIVE dataset, are compared with those of state-of-the-art. Se, Sp and Accuracy of our model is 0.8286, 0.9824 and 0.9689 respectively.

**Table 6:** Performance comparison of our proposed model on DRIVE dataset with other existing models.

Method	Year	Se	Sp	Acc	AUC
Ma *et al* [50]	2019	0.7916	0.9811	0.9570	0.9810
Guo *et al* [51]	2019	0.7891	0.9804	0.9561	0.9806
Wu *et al* [52]	2019	0.8038	0.9802	0.9578	0.9821
Wang *et al* [53] DU-Net	2019	0.7940	0.9816	0.9567	0.9772
Arsalan *et al* [39] VessNet	2019	0.8022	0.9810	0.9655	0.9820
Gu *et al* [54] CE-Net	2019	0.8309	-	0.9545	0.9779
Yin *et al* [48]	2020	0.8038	0.9837	0.9578	0.9846
Wang *et al* [49]	2020	0.7991	0.9813	0.9581	0.9823
Segnet-Basic [35]	2020	0.7949	0.9738	0.9579	0.9720
**Our Method**	2021	**0.8286**	**0.9824**	**0.9689**	**0.9825**

Similarly, results achieved from the implementation of our model on STARE dataset are compared in Table 7. From this experiment, Se, Sp and Accuracy of our model are 0.8238, 0.9866 and 0.9744 respectively.

**Table 7:** Performance comparison of our proposed model on STARE database with other existing models.

Method	Year	Se	Sp	Acc	AUC
Jin *et al* [47]	2019	0.8155	0.9752	0.9610	0.9804
Chen *et al* [55] Deeplab v3++	2018	0.8320	0.9760	0.9650	0.9735
Wang *et al* [53]	2019	0.8074	0.9821	0.9661	0.9812
Guo *et al* [51]	2019	0.7888	0.9801	0.9627	0.9840
Arsalan *et al* [39] VessNet	2019	0.8526	0.9791	0.9697	0.9883
Wu *et al* [52]	2019	0.8132	0.9814	0.9661	0.9860
SegNet-Basic [35]	2020	0.8118	0.9738	0.9543	0.9728
Wang *et al* [49]	2020	0.8239	0.9813	0.9670	0.9871
**Our Method**	2021	**0.8238**	**0.9866**	**0.9744**	**0.9877**

From the comparisons with state-of-the-art, it is obvious that our proposed model outperformed other existing models with respect to well-known evaluation metric accuracy on three well-known and publicly available datasets.

## 6 Conclusion

Diabetic retinopathy is one of the top ophthalmic diseases which lead to blindness in the diabetic patients. Accurate segmentation of retinal blood vessels significantly helps the ophthalmologist for screening and detection of diabetic retinopathy. In proceeding to the diagnosis of this disease, we proposed a segmentation network, DAVS-Net, for the segmentation of retinal blood vessels. Dense concatenation of features in the dense block enabled the network to acquire and transfer spatial information from the image. Fast convergence of the network is achieved through the edge information transfer from encoder layers to decoder layers. There are three main design attributes of DAVS-Net; Firstly, quality of features is improved by feature concatenation, whereas memory requirements are controlled by the bottleneck layers in dense block. Secondly, number of convolution layers is reduced in all six blocks of the network to minimize the spatial information loss. Thirdly, DAVS-Net employs dense paths for feature empowerment which aids in extraction of minor information from the image. We evaluated proposed network on three publicly available datasets and surpassed the existing state-of-the-art methods in terms of accuracy and computational efficiency. This method can be used as a second opinion system to aid medical doctors and ophthalmologists for the diagnosis and analysis of diabetic retinopathy. In the future, we will further increase the accuracy of blood vessels segmentation with consideration of other retinal diseases along.
